# SLEEP PROBLEMS MEDIATE THE ASSOCIATION BETWEEN CHILDHOOD ADVERSITY AND LATE-LIFE COGNITIVE FUNCTIONING

**DOI:** 10.1093/geroni/igad104.3086

**Published:** 2023-12-21

**Authors:** SangNam Ahn, Hong Xian, Jeffrey Scherrer, Asos Mahmood, Eunhye Ahn, Seonghoon Kim, McKenzie Beck, George Slavich

**Affiliations:** Saint Louis University, St. Louis, Missouri, United States; Saint Louis University, St. Louis, Missouri, United States; Saint Louis University, St. Louis, Missouri, United States; The University of Tennessee Health Science Center, Memphis, Tennessee, United States; Washington University in St. Louis, St. Louis, Missouri, United States; Singapore Management University, Singapore, Singapore; Saint Louis University, St. Louis, Missouri, United States; University of California, Los Angeles, Los Angeles, California, United States

## Abstract

The negative health impacts of childhood adversity have been well documented. However, little is known about the pathways linking childhood adversity with cognitive functioning later in life. We addressed this issue by examining the extent to which lifetime sleep problems mediated the effects of childhood adversity on late-life cognitive functioning. We used the 2002-2018 waves of the Health and Retirement Study in which participants were 20,607 adults (58.1% female), aged ≥50 years (Mage=68.9±9.5 years). Cognitive functioning was based on: 1) a 10-word immediate and delayed recall test of memory; 2) a serial 7s subtraction test of working memory; and 3) counting backwards to assess attention and processing speed. Sleep problems (e.g., difficulty initiating sleep) were assessed with the Jenkins Sleep Scale, and seven items of childhood adversity (e.g., physical abuse) were included from the list of lifetime potentially traumatic events. Path analysis revealed that there was a small but significant indirect relationship between childhood adversity and poor cognitive functioning through sleep problems (coefficient = -.014; p=.001). Indeed, about 3% of the effect of childhood adversity on cognitive functioning is mediated by sleep problems (i.e., 0.014 [indirect effect] / 0.476 [total effect]). Our findings can help policymakers and public health practitioners better understand the risk and protective factors of cognitive functioning later in life in relation to sleep problems among those individuals with childhood adverse events. Further research is warranted to determine if childhood adversity contributes to cognitive decline in those with trauma and PTSD compared to those with trauma alone.

